# Decoding the genetic basis of secretory tissues in plants

**DOI:** 10.1093/hr/uhae263

**Published:** 2024-09-16

**Authors:** Yuepeng Han

**Affiliations:** State Key Laboratory of Plant Diversity and Specialty Crops, Wuhan Botanical Garden of Chinese Academy of Sciences, Wuhan 430074, China; Hubei Hongshan Laboratory, Wuhan 430070, China

## Abstract

Although plant secretory tissues play important roles in host defense against herbivores and pathogens and the attraction of insect pollinators, their genetic control remains elusive. Here, it is focused that current progress has been made in the genetic regulatory mechanisms underpinning secretory tissue development in land plants. C1HDZ transcription factors (TFs) are found to play crucial roles in the regulation of internal secretory tissues in liverworts and *Citrus* as well as external secretory tissues in peach. C1HDZ TFs regulate secretory tissue development via synergistic interaction with AP2/ERF and MYC TFs. Thus, a set of genes are speculated to be recruited convergently for the formation of secretory tissues in land plants.

Most plants are able to secrete chemical compounds, such as salt, nectar, volatile oils, gums, latex and resins, from the cytoplasm to the cell exterior. Secreted compounds that are beneficial to plants are commonly termed secretions. Secretions are of special significance for plants to adapt to environmental stresses and to attract pollinators or defend against herbivores and pathogens [1, 2]. Hence, secretory tissues that are concerned with the secretion of beneficial compounds have figured prominently in considerations of the coevolution of plants and animals. Secretory tissues have a great diversity in the structure and topographic position. According to their position in the plant body, secretory tissues can be broadly classified into external and internal secretory tissues. The former is visually located in the outer surface of the epidermis, while the latter resides inside plant tissues and cannot be visualized externally.

External secretory tissues differentiate into three main types: glandular trichomes, nectaries, and hydathodes. Glandular trichomes are typical multicellular epidermal hairs that contain a secretory head borne on a stalk with an ability to exude specialized metabolites. Nectaries are composed of two categories, floral nectaries associated with flowers and extrafloral nectaries (EFNs) located on the vegetative parts. Both floral and extrafloral nectaries produce carbohydrate-rich nectar which connects the plant with its pollinators such as bees and defenders such as predatory insects [3]. Hydathodes are a class of permanently open pores at the leaf margins and tips that exude water as droplets. Like external secretory tissues, internal secretory tissues are classified into three main types: internal secretory cells, secretory cavities and ducts, and laticifers. Internal secretory cells, also known as idioblasts, are found dispersed in the normal cells, and most of them contain secretions. Secretory cavities and ducts are composed of lysigenous and schizogenous cavities. Lysigenous cavity is developed by the lysis of some cells filled with the secretory contents, while schizogenous cavity is a result of the separation of cells due to the breakdown of middle lamella but without the occurrence of cell lysis. Laticifers are fused tube-like cells filled with a milky fluid called latex. The different secretory structures are proposed to have evolved independently across multiple land plant lineages [1].

Many natural chemicals generated by plant secretory tissues have significant commercial value as fragrance ingredients, food additives, pharmaceuticals and pesticides. Significant advances have been achieved towards understanding the biochemical or ultrastructural aspects of secretory tissues [1]. Nevertheless, our knowledge about the molecular and genetic mechanisms regulating the development of secretory tissues is still limited. Glandular trichomes are the most common and studied external secretory structure as they can be found in nearly one-third of all vascular plants. Genes involved in glandular trichome development have been identified mainly in tomato and *Artemisia annua* [4, 5]. Tomato has different types of glandular and nonglandular trichomes on the leaf surface, while *A. annua* produces artemisinin in glandular trichomes which is a sesquiterpene lactone widely used for the treatment of malaria. Transcription factors (TFs) belonging to R2R3-MYB and HD-ZIP IV subfamilies have been demonstrated to play pivotal roles in glandular trichome initiation, while regulators associated with actin cytoskeleton and cuticle formation are crucial for glandular trichome morphogenesis [6]. Nearly all known genes involved in the development of glandular trichomes in *A. annua* and tomato have regulatory roles in non-glandular trichome development, supporting the hypothesis that glandular trichomes have evolved from non-glandular trichomes through the differentiation of apical cells into secretory cells [7]. Notably, trichomes and prickles share a certain degree of resemblance, but prickles are not modified trichomes. Genetically, trichomes and prickles are controlled by different gene networks [8]. This finding is confirmed by a recent study, where repeated co-option of *LOG* homologs associated with cytokinin biosynthesis was found to underlie prickle convergent evolution in numerous plant lineages [9].

Liverworts are among the earliest diverging plant lineages with adaptations to life on land, and extant ones diverged from extant angiosperms at one of the earliest nodes of land plant evolution. One clear-cut liverwort synapomorphy is the presence of oil bodies (OBs) that evolved as the first secretory tissues in land plants. OBs in liverworts accumulate a variety of specialized metabolites such as sesquiterpenoids and bisbibenzyls. Those chemicals are effective in protecting liverworts from arthropod herbivores and pathogens, but have cytotoxic effects on plant cells. Therefore, OBs are generally viewed as an adaptive strategy by which land plants accumulate chemicals with cytotoxic activity without autotoxicity. OBs in the complex thalloid liverworts (Marchantiopsida) are restricted to specialized idioblast cells. In recently years, there has been important advancement in understanding the mechanisms behind oil body formation in *Marchantia*. It is found that OBs in idioblastic cells are formed by redirecting the secretory pathway which is regulated at the transcription level [10]. *MpERF13* encoding an AP2/ERF protein and *MpC1HDZ* encoding a class I homeodomain leucine-zipper (HD-ZIP I) protein have been reported to play important regulatory roles in oil body formation in *Marchantia* [10, 11]. Deletion of *MpERF13* results in a complete loss of OBs, while loss-of-function mutations in *MpC1HDZ* cause a striking reduction in the number of OBs. Therefore, both MpERF13 and MpC1HDZ play a key role in mediating the differentiation of oil body cells.

Intriguingly, the paralogs of MpC1HDZ have been found to regulate the development of secretory tissues in angiosperms. In a study published two years ago in *Horticulture Research*, the researchers reported a candidate gene *PpLMI1* for the Mendelian *E* locus controlling extrafloral nectaries (EFNs) on peach leaf using the fine-scale mapping approach [12]. *PpLMI1* encodes a HD-ZIP I protein and it was named in line with its *Arabidopsis* ortholog LATE MERISTEM IDENTIFITY1 (LMI1). *PpLMI1* has a mutant that arose from an insertion of a 590-bp MITE-like transposable element of the hAT superfamily termed *mMoshan* in the third exon, thus, the coding region of the mutated transcript is 99-bp longer compared to the wild-type. In peach germplasm, leaf glands may be kidney-shaped (reniform), round-shaped (globose) or absent (eglandular). Genotyping of peach germplasm revealed that eglandular accessions are homozygous for the mutated allele, while reniform and globose accessions carry homozygous wild-type allele or both mutated and wild-type alleles, respectively. Like *Arabidopsis* LMI1, PpLMI1 is involved in the regulation of leaf margin structures in peach. Eglandular accessions produce deeply serrated leaves, while globose and reniform accessions have leaves with rounded and shorter crenellations. The association of deeply serrated leaves with the eglandular phenotype is consistent with the result that *PpLMI1* has significantly higher levels of expression in eglandular accessions than in both reniform and globose accessions. Thus, the mutated *PpLMI1* allele seems functional. Given the fact that the eglandular phenotype is associated with the high expression of *PpLMI1*, it is worthy of further study to clarify whether PpLMI1 regulates the development of peach EFNs via a dosage-dependent mechanism, by which a HD-XIP TF Woolly has been reported to specify fates of multicellular trichomes in tomato [4]. In addition, the eglandular peach cultivars are more susceptible to peach powdery mildew (*Podosphaera pannosa* var. *persicae*) than the reniform and globose cultivars. The positive influence of EFNs on powdery mildew resistance is in agreement with the view that EFNs are sugar-producing glands that endow plants with indirect defense against fungi and herbivores by attracting predatory insects such as ants [13]. Notably, EFNs are widespread in the leaf petiole of fruit tree species in *Prunus*, and the EFNs in this genus may be genetically controlled by the *LMI1* orthologs.

In *Citrus*, a PpLMI1 ortholog has been found to control the development of secretory cavities or oil glands in a study recently published in *Science* [14]. The aboveground organs of *Citrus* plants, particularly the leaf and fruit peel, have abundant oil glands that produce essential oils, a mixture of terpenoids, lipids and flavonoids, which are effective against herbivores. Forward genetic analysis of a natural kumquat mutant named ‘Hua Pi’ that specifically disrupts oil gland development reveals that a deletion of an 8.9-kb fragment containing *CsLMI1* is the causal mutation for the oil glandless trait. The regulatory role of *CsLMI1* in oil gland formation was confirmed by a CRISPR-Cas9-based knockout experiment, which displays several mutants with loss of *CsLMI1* function that exhibits the oil-glandless phenotype in all aboveground organs such as leaves. Interestingly, the *CsLMI1* promoter contains a conserved GCC box element, which recruits an AP2/ERF TF CsDRNL that was named following its *Arabidopsis* ortholog DORNRÖSCHEN-LIKE (DRNL). CRISPR-Cas9 editing of the GCC box causes a notable reduction in both *CsLMI1* expression and oil gland number, demonstrating the pivotal role of the GCC box for transcriptional activation. Knockout of *CsDRNL* by CRISPR-Cas9 causes a reduced but incomplete inhibition of *CsLMI1* expression. However, no oil glands are observed in the leaf tissues of CRISPR-edited *csdrnl* mutant. These results undoubtedly suggest that *CsDRNL*, like *CsLMI1*, is indispensable for oil gland formation in *Citrus*. Additionally, there is an association between oil gland formation and leaf serration in *Citrus*. Knockout of *CsLMI1* or *CsDRNL* can disrupt leaf serrations, resulting in a smooth leaf margin phenotype. Therefore, CsLMI1 and CsDRNL collaboratively participate in the regulation of leaf serration and oil gland development in *Citrus*. The synergism of HD-ZIP I and AP2/ERF TFs in secretory structure development has also been reported in liverworts, in which MpERF13 and MpC1HDZ are both required for oil body cell differentiation. However, whether MpC1HDZ acts downstream of MpERF13 in oil body development of liverworts remains elusive. Moreover, a bHLH TF CsMYC5, an *Arabidopsis* MYC5 ortholog, was identified to act downstream of CsLMI1 in oil gland development, affecting the formation of the sheath and epithelial cells of secretory cavities in *Citrus*. Although CsMYC5 has no regulatory role in leaf serration in *Citrus*, it is indispensable for essential oil biosynthesis. Likewise, an *Arabidopsis* MYC5 paralog termed PabHLH1 in the liverwort *Plagiochasma appendiculatum* has been reported to play a conserved regulatory role in terpenoid biosynthesis [15]. From these results it can be inferred that the AP2/ERF-C1HDZ-MYC pathway may be implicated in the regulation of OB cell differentiation in liverworts and EFN development in peach (Fig. 1).

**Figure 1:**
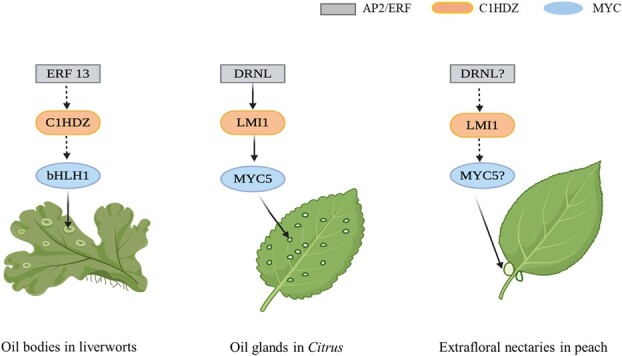
Schematic diagram for the AP2/ERF-C1HDZ-MYC pathway in the regulation of secretory tissues in liverworts, *Citrus*, and peach. DRNL, LMI1 and MYC5 in *Citrus* are paralogous to ERF3, C1HDZ and bHLH1 in liverworts, respectively. Oil glands in *Citrus* and extrafloral nectaries in peach are both genetically controlled by the orthologs of *Arabidopsis LMI1*, thus, their formation is probably regulated by similar gene networks. The dotted line indicates the possible relationship between TFs, while question marks indicate deduced genes that are associated with EFNs in peach.

From an evolutionary developmental perspective, identification of genes responsible for the regulation of secretory tissues is an essential step to trace the origin of secretory structures. The discovery of the association of the paralogs of liverwort OB-related TFs ERF13/C1HDZ/bHLH1 with secretory tissue development in *Citrus* and peach indicates that a set of genes might be recruited convergently for the formation of secretory tissues in land plants. This discovery also adds direct evidence to support the theory that oil bodies in liverworts evolved as the first secretory tissues in terrestrial plants [16]. Nectaries are widespread in floral and extrafloral positions in the plants. CRABS CLAW (CRC), a member of the YABBY TF family, is known to regulate floral nectary development in *Arabidopsis* and other core eudicot species [12]. CRC is also reported to regulate extrafloral nectary development in core eudicots based on the results of the correlation of *CRC* expression with the development of extrafloral nectaries on the midvein of leaves in *Gossypium* [17, 18]. However, forward genetic studies demonstrate that *GaNEC1* encoding a PB1 domain-containing protein is the causal gene for extrafloral nectary development in *Gossypium* [19]. Up to now, PpLMI1 is the only C1HDZ homolog known to control the development of EFNs in angiosperms, whereas, C1HDZ homologs have not been found to control floral nectary development in plants. These results, combined with the fact that nectaries occur in non-flowering plants, strongly support the hypothesis that the extrafloral nectaries precede the floral nectaries [1]. Ferns are the most prevalent non-flowering plants and bracken fern (*Pteridium aquilinum*) bear nectaries on the stem just below the leaf. Since the localization of nectaries in bracken fern is similar to that of EFNs in peach, it could be speculated that C1HDZ homologs are likely involved in the regulation of nectary development in bracken fern. In addition, the finding of different genes associated with nectary development is consistent with the concept that nectaries evolved multiple times independently during the course of plant evolution [20]. Identification of additional regulators governing the development of secretory tissues will provide new insights into the conservation and divergence of secretory tissues in plants.

## Data Availability

All data are available in the manuscript.

## References

[1] Fahn A . Secretory tissues in vascular plants. New Phytol. 1988;108:229–57 33873942 10.1111/j.1469-8137.1988.tb04159.x

[2] Richit JF, Díaz SVN, Dick LFP. et al. Neither lysigenous nor just oil: demystifying myrtaceous secretory cavities. Am J Bot. 2023;110:e16248 37792299 10.1002/ajb2.16248

[3] Roy R, Schmitt AJ, Thomas JB. et al. Review: nectar biology: from molecules to ecosystems. Plant Sci. 2017;262:148–64 28716410 10.1016/j.plantsci.2017.04.012

[4] Wu M, Chang J, Han X. et al. A HD-ZIP transcription factor specifies fates of multicellular trichomes via dosage-dependent mechanisms in tomato. Dev Cell. 2023;58:278–288.e5 36801006 10.1016/j.devcel.2023.01.009

[5] Yan T, Li L, Xie L. et al. A novel HD-ZIP IV/MIXTA complex promotes glandular trichome initiation and cuticle development in *Artemisia annua*. New Phytol. 2018;218:567–78 29377155 10.1111/nph.15005

[6] Chalvin C, Drevensek S, Dron M. et al. Genetic control of glandular trichome development. Trends Plant Sci. 2020;25:477–87 31983619 10.1016/j.tplants.2019.12.025

[7] Fahn A, Shimony C. Glandular trichomes of *Fagonia* L. (Zygophyllaceae) species: structure, development and secreted materials. Ann Bot. 1996;77:25–34

[8] Zhou N, Simonneau F, Thouroude T. et al. Morphological studies of rose prickles provide new insights. Hortic Res. 2021;8:221 34556626 10.1038/s41438-021-00689-7 PMC8460668

[9] Satterlee JW, Alonso D, Gramazio P. et al. Convergent evolution of plant prickles by repeated gene co-option over deep time. Science. 2024;385:eado1663 39088611 10.1126/science.ado1663 PMC11305333

[10] Kanazawa T, Morinaka H, Ebine K. et al. The liverwort oil body is formed by redirection of the secretory pathway. Nat Commun. 2020;11:6152 33262353 10.1038/s41467-020-19978-1 PMC7708844

[11] Romani F, Banić E, Florent SN. et al. Oil body formation in *Marchantia polymorpha* is controlled by MpC1HDZ and serves as a defense against arthropod herbivores. Curr Biol. 2020;30:2815–2828.e8 32559445 10.1016/j.cub.2020.05.081

[12] Lambert P, Confolent C, Heurtevin L. et al. Insertion of a mMoshan transposable element in PpLMI1, is associated with the absence or globose phenotype of extrafloral nectaries in peach [*Prunus persica* (L.) Batsch]. Hortic Res. 2022;9:uhab044 35039854 10.1093/hr/uhab044 PMC8829895

[13] Jones IM, Koptur S, von Wettberg EJ. The use of extrafloral nectar in pest management: overcoming context dependence. J Appl Ecol. 2017;54:489–99

[14] Wang H, Ren J, Zhou S. et al. Molecular regulation of oil gland development and biosynthesis of essential oils in *citrus* spp. Science. 2024;383:659–66 38330135 10.1126/science.adl2953

[15] Wu YF, Zhao Y, Liu XY. et al. A bHLH transcription factor regulates bisbibenzyl biosynthesis in the liverwort *Plagiochasma appendiculatum*. Plant Cell Physiol. 2018;59:1187–99 29528434 10.1093/pcp/pcy053

[16] Romani F, Flores JR, Tolopka JI. et al. Liverwort oil bodies: diversity, biochemistry, and molecular cell biology of the earliest secretory structure of land plants. J Exp Bot. 2022;73:4427–39 35394035 10.1093/jxb/erac134

[17] Lee JY, Baum SF, Oh SH. et al. Recruitment of *CRABS CLAW* to promote nectary development within the eudicot clade. Development. 2005;132:5021–32 16236772 10.1242/dev.02067

[18] Slavković F, Dogimont C, Morin H. et al. The genetic control of nectary development. Trends Plant Sci. 2021;26:260–71 33246889 10.1016/j.tplants.2020.11.002

[19] Hu W, Qin W, Jin Y. et al. Genetic and evolution analysis of extrafloral nectary in cotton. Plant Biotechnol J. 2020;18:2081–95 32096298 10.1111/pbi.13366 PMC7540171

[20] Morel P, Heijmans K, Ament K. et al. The floral c-lineage genes trigger nectary development in *petunia* and *Arabidopsis*. Plant Cell. 2018;30:2020–37 30087206 10.1105/tpc.18.00425 PMC6181019

